# Three-Week Old Pigs Are Not Susceptible to Productive Infection with SARS-COV-2

**DOI:** 10.3390/microorganisms10020407

**Published:** 2022-02-10

**Authors:** Elaine Haddock, Julie Callison, Stephanie N. Seifert, Atsushi Okumura, Tsing-Lee Tang-Huau, Shanna S. Leventhal, Matthew C. Lewis, Jamie Lovaglio, Patrick W. Hanley, Carl Shaia, David W. Hawman, Vincent J. Munster, Michael A. Jarvis, Juergen A. Richt, Heinz Feldmann

**Affiliations:** 1 Laboratory of Virology, Division of Intramural Research, National Institute of Allergy and Infectious Diseases, National Institutes of Health, Hamilton, MT 59840, USA; elaine.haddock@nih.gov (E.H.); callisonj@niaid.nih.gov (J.C.); stephanie.seifert@wsu.edu (S.N.S.); atsushi.okumura@nih.gov (A.O.); tsing-lee.huau@hotmail.com (T.-L.T.-H.); shanna.leventhal@nih.gov (S.S.L.); matthew.lewis@nih.gov (M.C.L.); david.hawman@nih.gov (D.W.H.); vincent.munster@nih.gov (V.J.M.); michael.jarvis@plymouth.ac.uk (M.A.J.); 2 Rocky Mountain Veterinary Branch, Division of Intramural Research, National Institute of Allergy and Infectious Diseases, National Institutes of Health, Hamilton, MT 59840, USA; jamie.lovaglio@nih.gov (J.L.); patrick.hanley@nih.gov (P.W.H.); carl.shaia@nih.gov (C.S.); 3 Faculty of Health, School of Biomedical Sciences, University of Plymouth, Plymouth, Devon PL48AA, UK; 4 The Vaccine Group Ltd., Plymouth, Devon PL48AA, UK; 5 Department of Diagnostic Medicine & Pathobiology, College of Veterinary Medicine, Kansas State University, Manhattan, KS 66506, USA; jricht@vet.k-state.edu; 6 Center of Excellence for Emerging and Zoonotic Animal Diseases, College of Veterinary Medicine, Kansas State University, Manhattan, KS 66506, USA

**Keywords:** SARS-CoV-2, young pigs, infection, replication, transmission, disease, pathology

## Abstract

As the COVID-19 pandemic moves into its third year, there remains a need for additional animal models better recapitulating severe COVID to study SARS-CoV-2 pathogenesis and develop countermeasures, especially treatment options. Pigs are known intermediate hosts for many viruses with zoonotic potential and are susceptible to infection with alpha, beta and delta genera of coronaviruses. Herein, we infected young (3 weeks of age) pigs with SARS-CoV-2 using a combination of respiratory and parenteral inoculation routes. Pigs did not develop clinical disease, nor macroscopic or microscopic pathologic lesions upon SARS-CoV-2 infection. Despite occasional low levels of SARS-CoV-2 genomic RNA in the respiratory tract, subgenomic RNA and infectious virus were never found, and SARS-CoV-2-specific adaptive immune responses were not detectable over the 13-day study period. We concluded that pigs are not susceptible to productive SARS-CoV-2 infection and do not serve as a SARS-CoV-2 reservoir for zoonotic transmission.

## 1. Introduction

The worldwide burden of the ongoing COVID-19 pandemic has increased to >397 million infections and >5.75 million deaths [1]. Since the discovery, countries have seen multiple COVID-19 waves caused by various SARS-CoV-2 variants of concern with increased transmissibility, enhanced virulence or immune evasion [2]. Vaccinations are administered worldwide as the key public health measure, but there is still an urgent need for better treatment options [3,4]. Animal models are pivotal for countermeasure development, but most current models are considered suboptimal for this purpose, with transient SARS-CoV-2 replication and only mild to moderate disease manifestation. Genetically modified rodent models have been associated with more severe, even lethal, disease and high viral loads, but sometimes displaying abnormal organ tropism affecting clinical presentation [5].

In the search for additional animal models, pigs generated an interest, in part because of their role as potential livestock amplification hosts with zoonotic potential, as evidenced for influenza A [6], Nipah [7] and Reston viruses [8]. Furthermore, pigs are susceptible to multiple alpha, beta and delta genera of coronaviruses, including: porcine respiratory coronavirus, which generally remains subclinical but can cause respiratory illness in conjunction with other pathogens [9]; porcine hemagglutinating encephalomyelitis virus, which causes vomiting, wasting and encephalomyelitis in young piglets [10]; as well as transmissible gastroenteritis virus, porcine epidemic diarrhea virus, porcine delta coronavirus and swine acute diarrhea syndrome coronavirus, all of these viruses mainly causing acute gastroenteritis in neonatal piglets [11]. Over the past year, several groups have investigated SARS-CoV-2 infection and replication in pigs [12,13,14,15,16,17]. Results from these studies differed in their fine detail, but in general, authors concluded that pigs are not susceptible to productive infection with SARS-CoV-2.

We started our work roughly at the same time as these other studies but focused on the use of young pigs (3 weeks of age) challenged with SARS-CoV-2 through a combination of multiple respiratory and parenteral routes. Low levels of genomic RNA were occasionally detected in mucosal swabs (oropharyngeal, nasal and rectal) in 7 of 8, and in lung tissue from 3 of 8 pigs, as well as in the tonsil, trachea and mediastinal lymph node of one animal each. However, pigs never developed any sign of clinical disease, nor macroscopic/microscopic pathology, and subgenomic RNA or infectious virus were not detected in any clinical specimen. There was also no unequivocal evidence for seroconversion within the two-week period following SARS-CoV-2 infection.

## 2. Materials and Methods

**Biosafety and animal welfare:** All infectious work with SARS-CoV-2 was performed in high biocontainment, in accordance with standard operating procedures approved by the Institutional Biosafety Committee (IBC) [18]. Animal work was approved by the Institutional Animal Care and Use Committee (IACUC) and was performed in strict accordance with national regulations. Pigs were group housed in pens and were fed commercial chow and water ad libitum. Pigs were monitored at least twice daily. Enrichment consisted of manipulanda, audio enrichment and human interaction. IACUC-approved endpoints were applied to determine when pigs were humanely euthanized [19,20].

**Virus:** The lineage A SARS-CoV-2 isolate nCoV-WA1-2020 (MN985325.1) was kindly provided by the Centers for Disease Control and Prevention, Atlanta, GA, USA as Vero passage 3 [21]. At RML, the virus was propagated one more time in Vero E6 cells to a virus stock titer of 2 × 10^6^ tissue culture dose 50 (TCID_50_). Sequence determination revealed no mutations (Genbank: MN985325.1) and no contaminants.

**Animal studies:** Commercially available Yorkshire cross piglets (male and female) were weaned and shipped at approximately 2 weeks of age. The pigs were obtained from the same farm as described by Meekins et al. [15]. Pigs were group housed in pens until the challenge age of 3 weeks. Eight pigs were randomly divided into two groups of four animals (*n* = 4). Pigs were inoculated with SARS-CoV-2 (1 × 10^5^ TCID_50_/mL) by the combined intranasal (1 mL in each nostril), oropharyngeal (3 mL), intratracheal (3 mL) and intravenous (2 mL) route; the total dose was 1 × 10^6^ TCID_50_ of SARS-CoV-2. After inoculation, pigs were monitored at least twice daily for signs of disease. On 0, 1, 3, 5, 7, 9, 11 and 13 days post infection (DPI), the animals were sedated for a physical examination. Bodyweight, SpO_2_, blood pressure, heart rate, respiration rate and temperature were measured; nasal, oropharyngeal and rectal swabs were collected to determine virus shedding. Thoracic radiographs were taken, and a venous blood sample was collected for hematology, clinical chemistry, serology and viremia. Four pigs were euthanized at 3 DPI; the remaining four were euthanized at 13 DPI. Full necropsies were performed on all pigs, including harvest of respiratory tract tissues (tonsil, oropharynx, trachea, right and left bronchus and six lung lobes), draining lymph nodes (cervical and mediastinal) and additional organs (heart, liver, spleen, kidney, stomach, jejunum, ileum and transverse colon) for gross pathology, histopathology and virology. Lungs were tested for PRRSV, influenza A virus, *Mycoplasma* spp. and bacterial ribosomal RNA.

**Thoracic radiographs.** Ventro-dorsal and right/left lateral radiographs were taken on clinical examination days prior to any other procedures. Radiographs were evaluated and scored for the presence of pulmonary infiltrates by two board-certified clinical veterinarians, according to a standard scoring system [19].

**Hematology and serum biochemistry analyses:** Hematology was completed on a Procyte DX (IDEXX Laboratories, Westbrook, ME, USA).

**Nucleic acid detection:** Nucleic acid extraction was performed according to approved protocols [18]. qRT-PCR was performed on RNA extracted from blood and swabs using QiaAmp Viral RNA kit (Qiagen, Hilden, Germany). Tissues (≤30 mg) were homogenized in RLT buffer, and RNA was extracted using the RNeasy kit (Qiagen). Viral RNA was detected with a one-step real-time RT-PCR assay (Quantifast, Qiagen) using primers and probes generated to target either genomic SARS-CoV-2 RNA (N gene) [22] or subgenomic SARS-CoV-2 RNA (E region [23]).

**Virus isolation:** Isolation was conducted on Vero E6 cells plated for 80% confluency in 48-well plates. Tissues were weighed in 1 mL DMEM with a 5mm stainless steel bead, homogenized at 30 Hz for 10 min and spun at 8000 rpm for 10 min. Homogenates were diluted 1:10 and 1:100 and used to infect Vero E6 cells in 0.1 mL DMEM, with an infection time of 45 min. Following inoculation, DMEM with 2.5% FBS, penicillin/streptomycin and L-glutamine was added for a total volume of 0.5 mL per well. Cell cultures were read for cytopathic effect.

**Histopathology:** Tissues were embedded in Pureaffin paraffin polymer (Cancer Diagnostics, Durham, NC, USA) and sectioned at 5 µm for hematoxylin and eosin (H&E) staining. For immunohistochemistry (IHC), tissues were processed using the Discovery Ultra automated stainer (Ventana Medical Systems) with a ChromoMap DAB kit (Roche Tissue Diagnostics cat#760-159). Specific immunoreactivity was detected using the GenScript U864YFA140-4/CB2093 NP-1 SARS-CoV-2-specific antiserum at a 1:1000 dilution. The secondary antibody was an anti-rabbit IgG polymer (cat# MP-6401) from Vector Laboratories ImPress VR. As all animals in this study were inoculated with SARS-CoV-2, uninfected tissues from previous age-matched porcine studies were used for control tissues.

**Antibody detection:** SARS-CoV-2-specific IgM and IgG responses were measured by enzyme-linked immunosorbent assay (ELISA). Plates were coated with 50 ng per well of antigen in DPBS for at least 1 h at room temperature or overnight at 4 °C. Antigens were obtained from Genscript: SARS-CoV-2 N protein (Catalog #Z03488), spike RBD (#Z03483) or S1 protein (#Z03501). Plates were washed with 5% skim milk in PBS + 0.05% Tween-20 and blocked with the same. Serum was diluted 1:100 in blocking buffer (5% skim milk in PBS + 0.05% Tween-20) and then applied to plates. Plates were washed with PBS + 0.05% Tween-20, and bound antibody was detected with either peroxidase-labeled rabbit anti-pig IgG (1:2000 dilution) (Invitrogen, Carlsbad, CA, USA) or goat anti-pig IgM (1:2000) (Bio-Rad Laboratories, Hercules, CA, USA). Plates were developed with ABTS 2-component solution (Seracare #5120-0032) and stopped with 5% sodium dodecyl sulfate (Sigma) in water. Absorbance at 405 nm was measured.

**T cell assay:** Peripheral blood mononuclear cells (PBMC) (5.0 × 10^6^) were seeded into duplicate wells in a 96-well flat-bottom plate, which was pre-coated with pig IFN-γ-capturing antibody (5.0 × 10^5^ cells/well-Pig IFN-γ Single-Color ELISPOT-ImmunoSpot^®^). PBMCs were stimulated with SARS-CoV-2 spike protein or SARS-CoV-2 nucleoprotein peptide pools at a final concentration of 2 µg/mL per peptide for 24 h. IFN-γ spots were developed and counted by CTL 328 ImmunoSpot^®^ Analyzer and ImmunoSpot^®^ Software.

**Statistical analysis:** Statistical significance was assigned when *p* values were <0.05 using Prism Version 8 (GraphPad). Paired Wilcoxon non-parametric test was applied for ELISpot, and two-way ANOVA with Sidak’s multiple comparisons test was used for ELISA.

## 3. Results

In this study, we used young 3-week-old pigs that were exposed to SARS-CoV-2 by a combination of respiratory (intranasal, oropharyngeal and intratracheal) and parenteral (intravenous) routes. The SARS-CoV-2 inoculum was back titrated to be 6.3 × 10^4^ TCID_50_/mL, amounting to a total dose of approximately 6 × 10^5^ TCID_50_ for each animal, which is close to the target dose of 1 × 10^6^ TCID_50_. Pigs were monitored daily for clinical signs and examined at predetermined timepoints. Four animals (#340-343) were necropsied at 3 DPI, representing the early phase of virus replication. Four additional animals (#344-347) were euthanized at 13 DPI, allowing for study of disease progression. Tissues were collected for pathology and virology at necropsy.

**Figure 1:**
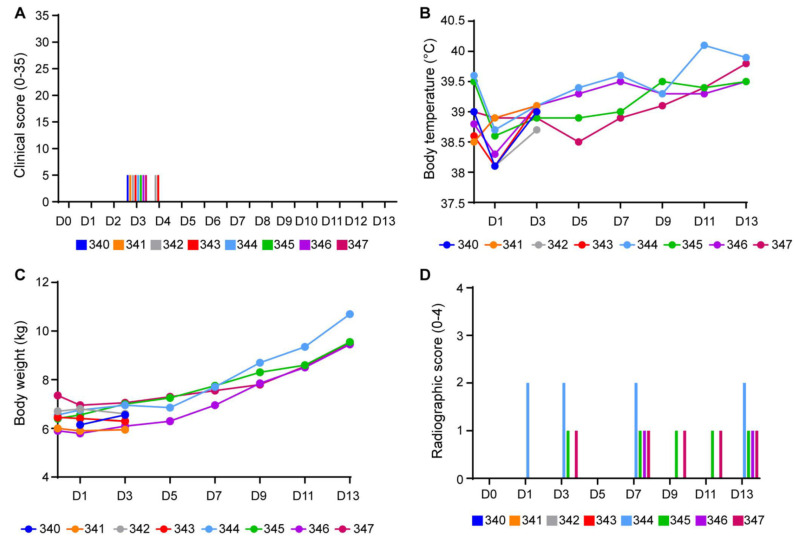
Clinical parameters of SARS-CoV-2 infection in 3-week-old pigs. Young pigs (3 weeks of age) were infected with SARS-CoV-2 by a combination of respiratory (intranasal, oropharyngeal and intratracheal) and parenteral (intravenous) routes (total dose 2 × 10^6^ TCID_50_)^6^ TCID_50_. Pigs were monitored daily for clinical signs until euthanasia on 3 DPI (#340–343) and 13 DPI (#344–347). The graphs display clinical scores (**A**), body temperature (**B**), body weight (**C**) and radiographic scores (**D**) for individual animals (the indicated color code is identical for animals in all figure parts).

**SARS-CoV-2 infection of 3-week-old pigs remained asymptomatic.** Clinical assessment was performed using a scoring system approved by the RML IACUC assessing general appearance, skin, nose/mouth/eyes, respiration, presence of any gross lesions and locomotion, as previously published [19,20]. All animals remained normal (score of 0) throughout the 13-day observation period, except for a low score of 5 at 2 DPI (all animals) and at 3 DPI (#342 and #343) (Figure 1A). The score of 5 reflected hyporeactivity when assessing locomotion, most likely due to two consecutive days of anesthesia (0 DPI and 1 DPI). Rectal temperatures were unremarkable, with only minor fluctuations noticed over the observation period of 13 days (Figure 1B). Body weight increased steadily, especially after 3 DPI, reflecting normal feeding behavior and growth for piglets at this young age (Figure 1C). Thoracic radiographs revealed very mild interstitial infiltrates (score 1 of 18) in one caudal lung lobe, starting at 3 DPI in the four animals that were euthanized at 13 DPI. One animal (#344) had mild pulmonary infiltrates in both caudal lung lobes (score 2 of 18) (Figure 1D). No other abnormalities were observed on the radiographs. Minor hematology changes were attributed to serial blood draws, and blood chemistry was unremarkable over the entire study period. Overall, SARS-CoV-2 infection did not cause overt disease in these young piglets.

**SARS-CoV-2 infection of 3-week-old pigs did not result in productive virus replication.** To determine the extent of virus replication in SARS-CoV-2-infected pigs (*n* = 8 for 0–3 DPI; *n* = 4 for 4–13 DPI)), qRT-PCR was performed on swabs (nasal, oropharyngeal and rectal), blood and organ tissue (Table 1). At 1 DPI, viral genomic RNA was detected in all three clinical swabs obtained from 5 of 8 pigs (#341, #342, #343, #346, #347), and viral genomic RNA was present in blood from a single animal (#343). However, by 3 DPI, viral genomic RNA was only detected in the rectal swab of one pig (#347), and later timepoints showed sporadic detection of low viral genomic RNA levels in oropharyngeal (#345, 7 DPI; #347, 9 DPI) and nasal (#344, 7 DPI) swabs (Table 1). Four pigs were euthanized at 3 DPI, and viral genomic RNA was detected in lung tissues of two of the animals (#342 and #343) and in the tonsil of a third animal (#341), all of which had positive swab samples at 1 DPI (Table 1). Of all the swabs collected from the remaining four pigs, only three swabs in total were positive for SARS-CoV-2 RNA between 5 DPI and 13 DPI (study endpoint), including oropharyngeal swabs collected from pig #345 at 7 DPI and pig #347 at 9 DPI and a nasal swab from pig #344 at 7 DPI (Table 1). At necropsy on 13 DPI, only 1 of 4 animals (pig #345) showed detectable viral genomic RNA in the lung (Table 1). This same animal also showed detectable viral genomic RNA in the trachea, and a second pig (#347) had detectable viral genomic RNA in the mediastinal lymph node. Additionally, of all swabs and tissues tested, no samples had detectable viral subgenomic RNA, and all attempts to recover infectious SARS-CoV-2 from clinical swabs and tissues from animals necropsied on 3 DPI and 13 DPI were unsuccessful. Overall, these results indicate that SARS-CoV-2 infection of 3-week-old piglets resulted in occasional low-level SARS-CoV-2 genomic RNA detection in respiratory tissues with no evidence of productive virus replication.

**Figure 2:**
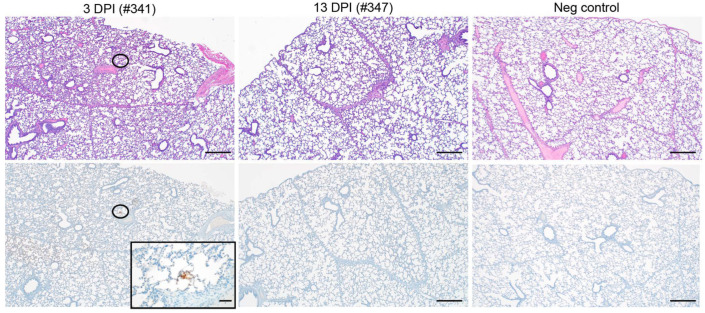
Pathology parameters of SARS-CoV-2 infected 3-week-old pigs. SARS-CoV-2 infected young pigs were euthanized on 3 DPI and 13 DPI, necropsied for tissue harvest and tissues prepared for histopathology. Negative control tissue samples were provided from previous age-matched studies. H&E did not find any SARS-CoV-2 related pathology, and IHC targeting SARS-CoV-2 nucleocapsid (N) protein remained negative. None of the pigs developed significant lung pathology, as shown here for one representative animal of the 3 DPI and 13 DPI. Except for a singular focus within a section of lung from pig #341 (3-DPI group), none of the lung samples showed SARS-2 immunoreactivity. The magnifications are indicated (40× and 400×).

**SARS-CoV-2 infection of 3-week-old pigs did not cause pathology.** None of the pigs euthanized at 3 or 13 DPI developed significant macroscopic or microscopic pathologic lesions (Figure 2, top panel). Likewise, except for a singular focus within a section of lung from pig #341 (3-DPI group) that showed small areas of SARS-CoV-2-specific antigen staining, none of the tissues sampled showed any SARS-CoV-2-specific immunoreactivity (Figure 2, bottom panel). Of note, two samples of cervical lymph nodes from the 13 DPI group exhibited a mild follicular hyperplasia. Acute hemorrhages were noted in a few lung sections from multiple pigs, which is believed to be an artifact caused by intracardiac euthanasia and/or necropsy. Within the lamina propria of the stomach, there were minimal infiltrating lymphocytes in seven of eight pigs, which is believed to be a background finding and common for the species. Likewise, four of eight pigs had a minimal lymphocytic enteritis or colitis, also believed to be incidental and common for the species. Any other findings noted in the remaining tissues were considered incidental and of no clinical relevance. Overall, SARS-CoV-2 infection did not cause relevant macroscopic/microscopic pathology in young pigs.

**Figure 3:**
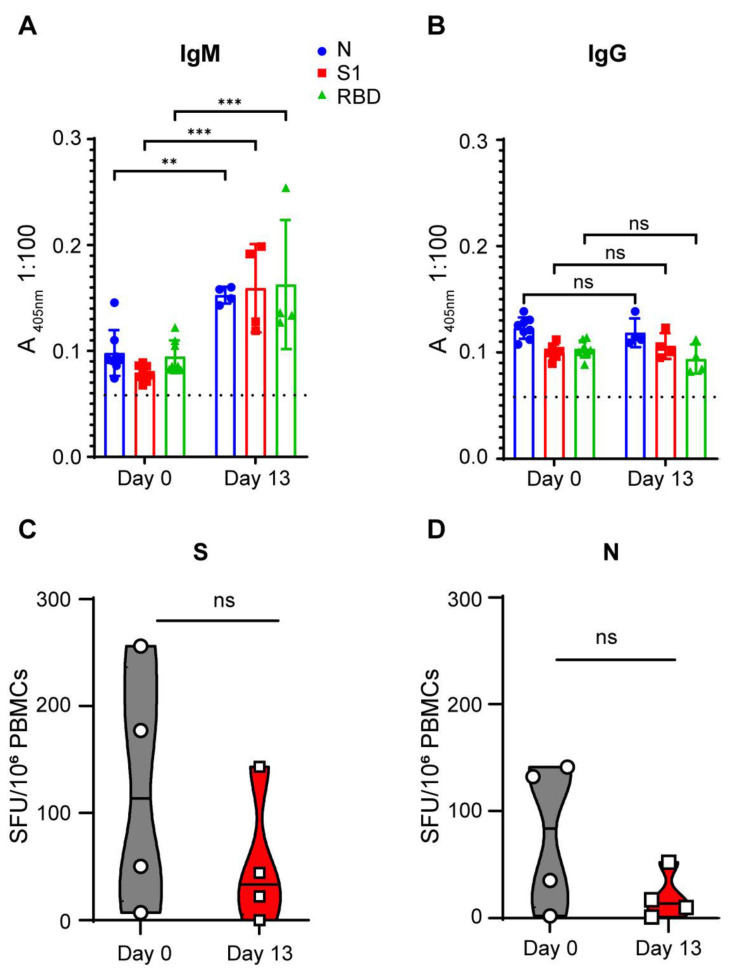
Immune responses to SARS-CoV-2 infection in 3-week-old pigs. Blood samples were collected on 0 DPI and 13 DPI to determine adaptive immune responses. (**A**,**B**) Humoral immune responses. Serum was analyzed for the presence of IgM (**A**) and IgG (**B**) antibodies directed against the nucleocapsid (N) protein, as well as the S1 subunit and the receptor-binding domain (RBD) of the spike (S) protein of SARS-CoV-2. Dashed line indicates mean background absorbance of wells receiving no serum. For statistical comparisons, we used a two-way ANOVA with Sidak’s multiple comparisons test (ns: *p* > 0.05, ** *p* < 0.01, *** *p* < 0.001). (**C**,**D**) Cellular immune responses. PBMCs were isolated and SARS-CoV-2 specific T cells were quantified by counting IFN-γ+ spot forming unit (SFU) following stimulation with a 15-mer SARS-CoV-2 S peptide pool (2 µg/mL) (**C**) or N peptide pool (2 µg/mL) (**D**). Individual SFU counts per 10^6^ stimulated PBMCs after deduction of background counts are shown. Mean SFUs ± SEM are plotted (*n* = 4; ns: not significant, Wilcoxon non-parametric test).

**SARS-CoV-2 infection of 3-week-old pigs did not mediate an adaptive immune response.** To determine whether SARS-CoV-2-infected pigs developed a humoral immune response to SARS-CoV-2 antigens, serum samples (1:100 dilution) collected prior to infection and at the end of the study (13 DPI) were subjected to IgM and IgG ELISAs targeting three different SARS-CoV-2 antigens: nucleocapsid (N), spike 1 (S1) and receptor binding domain (RBD) (Figure 3A,B). Against the three SARS-CoV-2 antigens, we observed statistically significant OD signal increases when comparing 0 DPI and 13 DPI sera for SARS-CoV-2-specific IgM (Figure 3A). The 13 DPI OD signal, however, was only borderline positive (mean 405 nm absorbance of <0.163 in 1:100 serum dilution), indicating that the IgM responses to SARS-CoV-2 antigens in the infected pigs were extremely low. Within the 13-day observation period, SARS-CoV-2-infected pigs did not generate a detectable SARS-CoV-2-specific IgG antibody response against the three SARS-CoV-2 antigens (Figure 3B). None of the pigs developed any detectable neutralizing antibodies against infectious homologous SARS-CoV-2 throughout the course of the study. T cell responses specific to SARS-CoV-2 S and N were assessed by a pig IFN-γ ELISPOT assay on PBMCs isolated before (D0) and after challenge (D13). We did not detect any increase in T cell response against the SARS-CoV-2 S and N antigens (Figure 3C,D). Overall, SARS-CoV-2 infection in young pigs did not generate an adaptive SARS-CoV-2-directed immune response.

## 4. Discussion

As a major livestock species, pigs cause concern as interim or amplifying hosts for viruses with zoonotic potential [6,7,8], and are susceptible to multiple coronaviruses [9,10,11]. Additionally, the pig ACE-2 receptor is able to bind to the RBD domain of the SARS-CoV-2 spike protein [24], and SARS-CoV-2 productively replicates in porcine cell lines [15], suggesting susceptibility and permissiveness of this livestock species to SARS-CoV-2 infection.

In the present study, SARS-CoV-2 infection of 3-week-old pigs resulted in the detection of occasional low-level SARS-CoV-2 genomic RNA in clinical samples and tissues mainly from the respiratory tract; however, neither viral subgenomic RNA could be detected nor infectious virus could be isolated from these SARS-CoV-2 genomic RNA-positive specimens. This is consistent with other studies that found slightly older pigs to be non- or only weakly permissive to SARS-CoV-2 [12,13,14,15,16,17]. None of these pig studies, including the present one, have therefore been able to unequivocally show SARS-CoV-2 replication at any significant level that would lead to disease manifestation or pathology. This has now been shown even for young piglets, with studies spanning pigs from 3 to 9 weeks of age at time of experimental infection [12,13,14,15,16,17]. Thus, unless adult pigs are more susceptible and permissive to SARS-CoV-2, domestic pigs do not represent a valuable SARS-CoV-2 disease or infection model. If explored any further, studies in adult domestic pigs would be challenging for most biocontainment facilities due to animal size and weight, but miniature pigs might be an alternative option.

At least one other study [16] reported occasional detectable SARS-CoV-2 genomic RNA in the upper respiratory tract, similar to what we have reported here. However, viral RNA loads seem insufficient to support biologically relevant virus shedding and thus interspecies or intraspecies transmission. This is supported by the lack of virus isolation from upper respiratory tract clinical material. Thus, we conclude that young pigs are unlikely to be of significant concern as carriers of SARS-CoV-2, nor a concern for zoonotic SARS-CoV-2 transmission.

Interestingly, we report here that SARS-CoV-2 genomic RNA was recovered from lung tissue in 3 of 8 pigs and rare SARS-CoV-2-specific antigen staining in the lung of one pig. However, the lack of viral subgenomic RNA detection, virus isolation and lung pathology question the biological relevance of this finding that has not been reported in any other SARS-CoV-2 pig study. It is likely that the presence of SARS-CoV-2 genomic RNA represents residual input virus and that a potent innate immune response quickly controlled SARS-CoV-2 infection in pigs.

Three previous studies [12,13,15] did not report convincing SARS-CoV-2-specific humoral immune responses, especially after virus delivery to the respiratory tract. Two studies, however, reported seroconversion after parenteral virus delivery [16,17], and another study after oronasal infection [16]. In our study, we found an equivocal, borderline IgM antibody response two weeks after SARS-CoV-2 infection, but no evidence of IgG antibodies, nor for T-cell-mediated immune responses, and thus no unequivocal specific adaptive immune response to SARS-CoV-2 exposure through combined respiratory and parenteral routes. Unfortunately, we were unable to extend our study for an additional time period due to weight restrictions for our biocontainment facility. Studies in other animal models (hamster, rhesus macaque), however, showed adaptive immune response in less than one week of infection [5,25].

## 5. Conclusions

Across the different SARS-CoV-2 pig studies [12,13,14,15,16,17], there are multiple variable parameters, such as pig breed, age at challenge, SARS-CoV-2 challenge strain/variant and challenge dose, and routes of exposure that may have influenced differences in outcome. Overall, however, the differences are minor, and there is no convincing evidence that pigs get productively infected with SARS-CoV-2. We conclude that even young piglets are neither a suitable COVID-19 animal model nor an important carrier of SARS-CoV-2. Together, these results indicate that pigs do not represent a concern for zoonotic/reverse zoonoticSARS-CoV-2 transmission. Furthermore, it seems unlikely that SARS-CoV-2 infection of pigs is a veterinary or food safety concern.

## Figures and Tables

**Table 1:** SARS-CoV-2 genome copy numbers in blood, swabs and tissues. DPI = days post infection (for tissues, 3 means 3 DPI and applies to animals #340–343, 13 means 13 DPI and applies to animals #344–347; Oroph. = oropharyngeal; LN = lymph node; RUL = right upper lobe; RML = right medium lobe; RLL = right lower lobe; LLL = left lower lobe; LML = left medium lobe; LUL = left upper lobe; n/a = not applicable (animal euthanized on 3DPI); − = negative; numbers = copy numbers/mL (swabs and blood) or copy numbers/g (tissues) of SARS-CoV-2 N segment. Tissues not listed were found to be negative for genomic N RNA, and all tissues tested were negative for subgenomic E RNA.

Sample	DPI	#340	#341	#342	#343	#344	#345	#346	#347
**Oroph. swab**	0	-	-	-	-	-	-	-	-
	1	-	-	**10,543**	-	-	**-**	**3600**	-
	3	-	-	-	-	-	-	-	-
	5	n/a	n/a	n/a	n/a	-	-	-	-
	7	n/a	n/a	n/a	n/a	-	**1543**	**-**	**-**
	9	n/a	n/a	n/a	n/a	-	**-**	**-**	**2743**
	11	n/a	n/a	n/a	n/a	-	**-**	**-**	**-**
	13	n/a	n/a	n/a	n/a	-	**-**	**-**	**-**
**Nasal swab**	0	-	-	-	-	-	**-**	**-**	**-**
	1	-	-	**22,029**	**2486**	-	**-**	**-**	**3247**
	3	-	-	-	-	-	-	-	-
	5	n/a	n/a	n/a	n/a	-	-	-	-
	7	n/a	n/a	n/a	n/a	**10,886**	-	-	-
	9	n/a	n/a	n/a	n/a	-	-	-	-
	11	n/a	n/a	n/a	n/a	-	-	-	-
	13	n/a	n/a	n/a	n/a	-	-	-	-
**Rectal swab**	0	-	-	-	-	-	-	-	-
	1	-	**1738**	-	-	-	-	-	**6982**
	3	-	-	-	-	-	-	-	**2196**
	5	n/a	n/a	n/a	n/a	-	-	-	-
	7	n/a	n/a	n/a	n/a	-	-	-	-
	9	n/a	n/a	n/a	n/a	-	-	-	-
	11	n/a	n/a	n/a	n/a	-	-	-	-
	13	n/a	n/a	n/a	n/a	-	-	-	-
**Blood**	0	-	-	-	-	-	-	-	-
	1	-	-	-	**3491**	-	-	-	-
	3	-	-	-	-	-	-	-	-
	5	n/a	n/a	n/a	n/a	-	-	-	-
	7	n/a	n/a	n/a	n/a	-	-	-	-
	9	n/a	n/a	n/a	n/a	-	-	--	-
	11	n/a	n/a	n/a	n/a	-	-	-	-
	13	n/a	n/a	n/a	n/a	-	-	-	-
**Lung RUL**	13	-	-	-	-	-	-	-	-
**Lung RML**	13	-	-	**51,000**	**-**	**-**	**18,333**	-	-
**Lung RLL**	13	-	-	**-**	**53,333**	**-**	**-**	-	-
**Lung LUL**	13	-	-	**48,667**	**26,667**	**-**	**-**	-	-
**Lung LML**	13	-	-	-	-	-	-	-	-
**Lung LLL**	13	-	-	-	-	-	-	-	

## Data Availability

All data are shown in the manuscript. Additional requests should be directed to the corresponding author.
